# Spontaneous Left Main and Right Coronary Artery Spasm in a Patient With Vasospastic Angina

**DOI:** 10.1177/2324709617732796

**Published:** 2017-09-26

**Authors:** Analkumar Parikh, Thomas Paul Vacek

**Affiliations:** 1 Wright State University, Dayton, OH, USA

**Keywords:** coronary spasm, angina, cardiology, calcium channel blocker, nitroglycerin, interventional

## Abstract

Coronary spasm is a well-documented, though rare, condition that can mimic myocardial infarction and is usually found in only a single vessel during an event. We describe the case of a 43-year-old male with past medical history of hypertension, hyperlipidemia, tobacco abuse, and with no known coronary disease. The patient developed chest pain 3 days postadmission for primary diagnosis of psychiatric disorder. The patient had a positive stress study with moderate reversible ischemia in the anterolateral region. A subsequent coronary angiography was performed that revealed significant left main coronary obstruction with TIMI I (thrombolysis in myocardial infarction) flow. This pattern was also present in the proximal right coronary artery. Both stenoses were relieved with intracoronary nitroglycerin, revealing no significant obstructive disease and TIMI III flow. The patient was started on dihydropyridine calcium channel blocker and counseled against smoking without recurrence of angina.

## Background

Coronary artery spasm (CAS) causing supply and demand mismatch can mimic coronary ischemia, resulting in unnecessary interventions. Among the few reported cases of left main CAS, most descriptions are due to catheter-based irritation of the vessel. However, bilateral spontaneous coronary artery vasospasm of the right and left main coronary artery is an exceptionally rare occurrence in a single patient and during the same procedure. We describe a patient who had a strong clinical history consistent with vasospastic angina that demonstrated spasm of left main and proximal right coronary artery.

Coronary artery spasm is reported to occur in 1% to 5% of percutaneous coronary interventions, and the mechanism is thought to be secondary to mechanical disturbance from guide wire insertion. This can sometimes produce dramatic fatal consequences, such as cardiogenic shock, as well as malignant ventricular arrhythmia.^1^

## Case Presentation

The patient was a 43-year-old male who was admitted to the psychiatry ward from an outside hospital for further management of homicidal and suicidal ideation. The patient developed chest pain 3 days postadmission. On further questioning, he gave a history of frequent and recurrent chest pain symptoms over the past 1 year. These episodes were associated with substernal chest pressure, which had an aching quality. Physical examination was otherwise normal. His past medical history was significant for hypertension, hyperlipidemia, tobacco use, and polysubstance abuse.

**Figure1.**
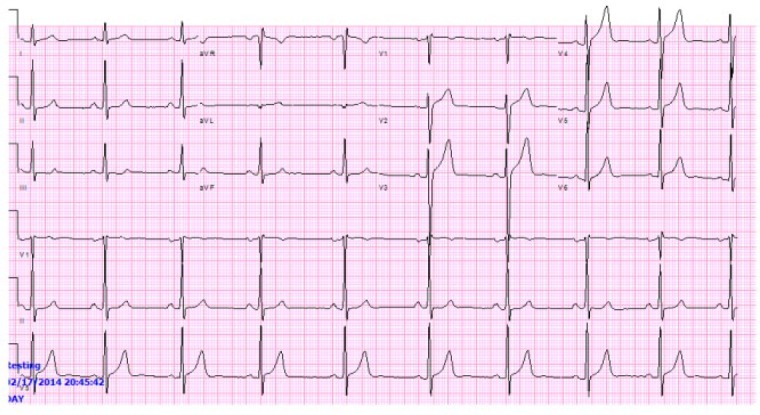
Initial presenting EKG without acute ST abnormality.

On initial evaluation, the electrocardiogram (EKG; Figure 1) showed sinus rhythm without acute ST changes. The laboratory evaluation was within normal limits, including the initial cardiac enzymes. He underwent a myocardial perfusion imaging study that showed moderate reversible ischemia in the anterolateral region, suggestive of significant ischemia. Echocardiography examination was without wall motion abnormalities.

**Figure 2.**
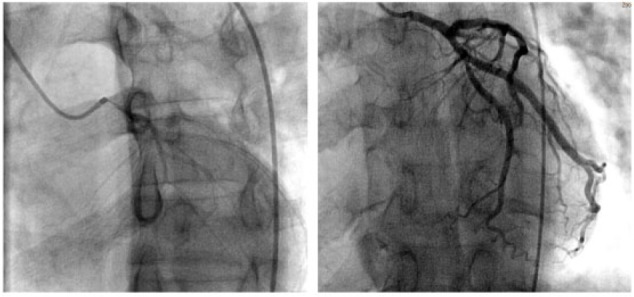
Image on the left shows spasm of the left main with TIMI I flow of left anterior descending (LAD) and left circumflex (LCFX) arteries. Image on the right shows resolution of spasm with TIMI III flow after intracoronary nitroglycerin administration. There was no significant obstructive disease in the LAD or LCFX arteries.

**Figure 3.**
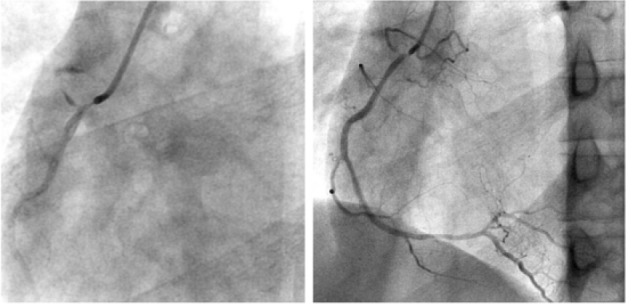
Image on the left shows spasm of the proximal portion of the right coronary artery with TIMI I flow and resolution of spasms with intracoronary nitroglycerin administration with return to TIMI III flow and no significant obstructive disease.

Subsequently, coronary angiography (Figures 2 and 3) was performed, and the initial left coronary injections revealed significant left main coronary narrowing. The left anterior descending and circumflex coronary arteries were without any significant lesions. The right coronary artery angiogram revealed presence of severe spasm in the proximal segment with appropriate response to 200 µg intracoronary nitroglycerin. Subsequently, the left main coronary artery was reengaged and another dose of 200 µg of nitroglycerin was given intracoronary. On the repeat left coronary angiogram, the left main stenosis due to coronary vasospasm was resolved. The patient was started on calcium channel blockers, counseled on smoking cessation, and has not yet had recurrence of angina.

## Discussion

Coronary artery spasm is typically associated with other substances and circumstances including alcohol, marijuana, amphetamine, chemotherapeutic agents, antimigraine therapy, physical stress with activation of autonomic nervous system, and magnesium deficiency.^2,3^ CAS was especially prominent among smokers (4.2 increased incidence) and those patients with hyperlipidemia (2.3 increased incidence).^4,5^ Studies suggest that polymorphisms of endothelial NO synthetase in low-grade inflammation are thought to be the most important risk factors for vasospastic angina.^6^ Moreover, genetic alleles were associated with increased incidence of CAS and smooth muscle cell hyperreactivity.^7^ The polymorphisms found were from genes of endothelial NO synthase gene, NADH/NADPH oxidase p22 phox gene, Stromelysin-1, and interleukin-6 genes; many of these genes are involved in inflammatory pathways and NO handling.^3^

Establishing the diagnosis is not easy since spasm can be a brief episode. EKG results are useful as well as ambulatory monitoring or exercise testing, though they do not always capture the events. CAS can sometimes be present even without symptoms and also not be established by any of these tests. Coronary angiography with provocative testing is the gold standard for diagnosing CAS if one cannot establish diagnosis via other short-term monitoring, long-term monitoring, or exercise provocation.^8^ Ergonovine and acetylcholine have been some agents used in the catheterization laboratory to help provoke spasm when it cannot be captured otherwise.^8^ However, a negative angiographic provocative test also does not definitively exclude CAS as cause of angina.

Our patient suffered from severe episodes of chest pain, thought to be caused by inadequate coronary blood flow. At angiography, he appeared to have severe stenosis of left main and right coronary artery, which improved with intracoronary nitroglycerin injection. The cause of the CAS in this patient is uncertain. This could have resulted from the injection of contrast medium due to mechanical irritation by tip of the catheter or a spontaneous phenomenon. The obvious clinical differentiation of coronary spasm from fixed obstructive disease has significant clinical implications.

Current antivasospastic therapy of choice is calcium channel blockers (either dihydropyridine or nondihydropyridine) and/or nitrates.^2^ Beta-blockers are contraindicated as beta blockade may cause unopposed alpha-adrenergic stimulation and increased vasoconstriction. It has been noted that multiple calcium channel blockers were necessary to relieve angina. The final addition of felodipine to diltiazem, isosorbide mononitrate, and aspirin produced a resolution of angina in at least one patient.^2^ Drug-refractory CAS occurs in approximately 20% of patients with CAS.^9^ Other procedures have been tried such as balloon angioplasty, percutaneous coronary intervention with stents, and coronary artery bypass with limited success.^10,11^

## Conclusion

Bilateral right coronary and left main CAS is uncommon and should be kept in mind as a possibility. Left main and right CAS was relieved only following administration of intracoronary nitroglycerin. The vasospasm of the coronary arteries may be spontaneous or iatrogenic (catheter-induced). Vasospasm may be considered in differential diagnosis particularly when angiography reveals no significant atherosclerosis disease in other coronary arteries.
